# Subgenomic Distribution and Herbicide Cross‐Resistance of *ALS* Gene Mutations in Allohexaploid *Echinochloa crus‐galli*

**DOI:** 10.1002/pld3.70168

**Published:** 2026-04-21

**Authors:** Luan Cutti, Guilherme Menegol Turra, Filipi Mesquita Machado, Estéfani Sulzbach, Paula Sinigaglia Angonese, Catarine Markus, Todd A. Gaines, Aldo Merotto

**Affiliations:** ^1^ Crop Science Department Federal University of Rio Grande do Sul Porto Alegre Rio Grande do Sul Brazil; ^2^ Department of Agricultural Biology Colorado State University Fort Collins Colorado USA

**Keywords:** barnyardgrass, cross‐resistance, polyploid, subgenome, target site resistance

## Abstract

Herbicide target site resistance in polyploid species is more complex than in diploids due to potential subgenome interactions. This study characterized mutations in the *ALS* gene across distinct subgenomes of hexaploid *Echinochloa crus‐galli* and evaluated the cross‐resistance patterns conferred by each mutation to various ALS‐inhibiting herbicides. *E. crus‐galli* populations were screened, and dose–response curves were performed with ALS inhibitors from different chemical groups. The *ALS* gene copies of each subgenome (A, B, and C) were sequenced. Copy number variation, global relative expression, and the specific relative expression of *ALS* gene from each subgenome were performed. The mutations Ala122Thr, Ala205Asn, and Ser653Asn conferred resistance only to imazethapyr, whereas Trp574Leu to imazethapyr, penoxsulam, bispyribac‐sodium, and nicosulfuron, when considered the label rate. *ALS* mutations were more frequent in subgenome A, but *ALS* from subgenome C had the highest expression. Biotypes with the same mutation showed different resistance level to herbicides. The biotype SAOJER‐01 had Trp574Leu mutation in subgenome C and was 22 times more resistant to imazethapyr and penoxsulam than CAMAQ‐01, which had the same Trp574Leu mutation in subgenome A. Both SAOJER‐01 and CAMAQ‐01 biotypes showed CYP450 metabolism mediating penoxsulam resistance in addition to the target site mutation. In conclusion, the mutations Ala122Thr, Ala205Asn, Trp574Leu, and Ser653Asn confer resistance to imazethapyr, but only Trp574Leu confers resistance to the other chemical groups. The herbicides penoxsulam, bispyribac‐sodium, and nicosulfuron are effective in controlling three out of four mutations. CYP450‐mediated metabolism coexists in biotypes carrying the Trp574Leu mutation. The subgenome location of the *ALS* mutation may result in variable levels of resistance.

## 1 Introduction

Herbicide resistance is one of the major threats to crop protection and food safety. Weeds have been identified as resistant to several herbicide modes of action through several resistance mechanisms. The herbicide resistance mechanisms are classified into two major categories: target site and nontarget site (Mucheri et al. 2024). Target site mechanisms refer to any alteration in the enzyme inhibited by the herbicide molecule that affects the binding or the overproduction of the target enzyme due to increased expression or increased number of the coding gene across the genome (Murphy and Tranel 2019). Nontarget site is a more complex character and involves different strategies utilized by plants, including increased capacity to metabolize the herbicide, usually driven by *CYP*, *UGT*, *GST*, or *AKR* genes upregulation, and reduced translocation by vacuolar sequestration, related to ABC transporters (Jugulam and Shyam 2019). Depending on the mechanism of resistance, cross‐resistance can occur to other herbicide molecules that target the same enzyme. For example, the Trp574Leu mutation in the *acetolactate synthase* (*ALS*) gene is well known to confer resistance to all ALS inhibitors in many different species (Gherekhloo et al. 2018; Wang et al. 2019). The same mutation may confer different resistance levels when compared in two different species or even may confer different cross‐resistance patterns, as shown when comparing the effect of Pro197Thr in *Papaver rhoeas* and *Bromus japonicus* (Kaloumenos et al. 2011; Liu et al. 2024). The explanation for the different phenotypic expression of the same mutation may be related to genome biology, such as ploidy level and subgenome interactions, or to an additional nontarget site resistance mechanism.

Polyploidy is more frequent than diploidy in plants (te Beest et al. 2012), although this characteristic is not considered in several studies about herbicide resistance. Diploids are those with the genome composed of two sets of chromosomes of the same subgenome, whereas polyploids are those with three, four, five, or six chromosome sets called triploid, tetraploid, pentaploid, or hexaploid, respectively (Doyle et al. 2008; Heslop‐Harrison et al. 2023). Allopolyploids are polyploids that had the genome duplication event originating from an interspecific crossing, whereas autopolyploids result from duplication of the same genome within a species (Doyle et al. 2008). Crops such as wheat (allotetraploid and allohexaploid varieties), oat (allohexaploid), cotton (allotetraploid), and potato (auto‐allotetraploid and allopentaploid) are examples of polyploids (Akagi et al. 2022). However, the plant kingdom also includes polyploid troublesome weeds, such as *Echinochloa crus‐galli* (allohexaploid), *E. colona* (allohexaploid), *E. phyllopogon* (allotetraploid) (Wu et al. 2022; Ye et al. 2020), *Poa annua* (allotetraploid) (Robbins et al. 2023), *Leptochloa chinensis* (tetraploid) (Wang et al. 2022), and *Avena fatua* (allohexaploid) (Yang et al. 1999). The consequences of polyploidization are the accumulation of redundant alleles of the same gene, making them available to new functionalization or to combine efforts to play together on certain characteristics. When polyploidization and herbicide resistance are combined, the main consequences may be the increased number of metabolism and herbicide target coding genes, which may favor faster and more complex evolution of herbicide resistance in comparison with diploid weeds. *E. crus‐galli* is an allohexaploid species, carrying two chromosome sets from three different subgenomes (AABBCC), a total of 54 chromosomes (2n = 6x = 54), and has been successful at invading different crop fields worldwide (Wu et al. 2022), with rapid herbicide resistance evolution to several herbicides due to target and nontarget site mechanisms (Pan et al. 2022; Turra et al. 2023). The *E. crus‐galli* weediness may be favored by being polyploid, for example, the presence of 867 *CYP*, 227 *GST*, 361 *ABC*, and 113 *AKR* genes in its genome may favor the evolution to metabolic resistance when compared to *E. haploclada* , a diploid species with many less genes and much less agricultural impact (Wu et al. 2022; Ye et al. 2020). The herbicide target site coding genes are also amplified in polyploids; for example, in *E. crus‐galli* , there are three *ALS* genes (Panozzo et al. 2021) and six *ACCase* genes (Iwakami et al. 2024). The impact of polyploidization on herbicide target site resistance warrants further study.

ALS inhibitors are herbicides utilized at very low rates that have a favorable toxicity profile and are efficient in controlling a wide range of weed species while being selective to many crops, such as rice, wheat, corn, and soybean (Zhou et al. 2007). Consequently, the ALS‐inhibiting herbicide mode of action has the most resistance cases reported (Heap 2024) and the most target site resistance investigation studies. To date, mutations in nine *ALS* positions have been reported, including Ala122, Pro197, Ala205, Phe206, Asp376, Arg377, Trp574, Ser653, and Gly654. The most common mutations are Trp574Leu and Pro197 to a range variety of amino acids (Gaines et al. 2020; Heap 2024). The ALS‐inhibiting herbicides are sorted into seven chemical groups according to HRAC (Herbicide Resistance Action Committee) 2024 classification: imidazolinones, sulfonylureas, triazolopyrimidines—type 1, triazolopyrimidines—type 2, pyrimidinylbenzoates, triazolinones, and sulfonanilides. Considering the favorable chemistry profile of ALS inhibitors combined with the scenario where *E. crus‐galli* resistant has worldwide distribution in crops with limited options for crop‐selective herbicides, the knowledge of cross‐resistance provided by target site mutations could lead to a rationale rotation with different chemical groups from ALS inhibitors, helping to utilize this valuable mode of action. In addition, diving into the weed genomics era and the knowledge about the presence of different alleles in polyploid species must be considered when studying target site mutations. The current effort to generate genomic resources for weed species (Montgomery et al. 2024) and a deeper understanding of genome biology raises the question about the unknown effects of mutations located in different subgenomes in polyploid weeds and the consequences for herbicide resistance. This study aimed to characterize mutations in the *ALS* gene across distinct subgenomes of allohexaploid *E. crus‐galli* and to evaluate the cross‐resistance patterns conferred by each mutation to various ALS‐inhibiting herbicides.

## 2 Material and Methods

### 2.1 Population Collections and ALS Inhibitor Screening

Seeds from 100 *E. crus‐galli* populations were collected in Southern Brazil, mainly Rio Grande do Sul state between 2011 and 2020. The seeds were germinated and at 3–4 leaf stage screened with four active ingredients from different chemical groups of ALS inhibitors herbicides: imazethapyr (imidazolinones), penoxsulam (triazolopyrimidine—type 2), bispyribac‐sodium (pyrimidinylbenzoates), and nicosulfuron (sulfonylureas). The doses sprayed were the label rate of each herbicide, 106 g ha^−1^ of imazethapyr (Imazetapir Plus Nortox, Nortox, 106 g L^−1^) + adjuvant Dash (BASF) 0.5% v/v, 60 g ha^−1^ of penoxsulam (Ricer, Corteva, 240 g L^−1^) + adjuvant Veget'Oil (Oxiquímica, 0.5% v/v), 50 g ha^−1^ of bispyribac‐sodium (Nominee 400 SC, Iharabras, 400 g L^−1^) + adjuvant Dash (BASF) 0.5% v/v, and 60 g ha^−1^ of nicosulfuron (Nicosulfuron Nortox 40 SC, Nortox, 40 g L^−1^), at spray volume of 200 L ha^−1^. The application was performed with an automated spray chamber (Generation III, DeVries Manufacturing). The screening was conducted in a greenhouse (28°C ± 5, supplemented with 500–600 μmol m^−2^ s^−1^ in a 14/10 h [light/dark] cycle), and the plants were kept flooded during the duration of the experiment. Each treatment had four replicates. The control efficacy was assessed at 28 days after treatment (DAT), where 0% indicated no injury and 100% indicated dead plants. Plants with up to 80% injury while remaining alive were considered resistant.

### 2.2 Subgenome Location of *ALS* Mutations

To identify possible target site mutations, the DNA of three individuals from 19 populations from the initial screening experiment had the *ALS* gene partially amplified utilizing the primers flanking the known positions where mutations have been previously identified (Table 1), following the conditions described in Turra et al. (2023). These primers target the *ALS* gene of all three *E. crus‐galli* subgenomes. To sequence the individual *ALS* genes of each subgenome, the PCR product of one individual of each biotype was inserted into a vector and then transformed in *E. coli* utilizing the TOPO TA Cloning Kit for Sequencing, with One Shot TOP10 Chemically Competent *E. coli* (Invitrogen), following the manufacturer's protocol. The plates with colonies were incubated for 24 h at 37°C. Twelve individual colonies of a single individual of each biotype were isolated to perform a colony‐PCR utilizing the primers targeting the position where the mutation was previously identified, and the PCR product was Sanger sequenced. The distinction of the three *ALS* genes was based on single nucleotide polymorphisms (SNPs) between them, comparing the Genbank sequences MH013494.1 (*ALS*‐SubA), MG188321.1 (*ALS*‐SubB), and MH013493.1 (*ALS*‐SubC) (Figure S1) and the 12 colonies sequenced of each individual from each biotype. The subgenome was attributed to each sequence by comparing the three Genbank sequences with the *ALS* genes of the last version of *E. crus‐galli* genome (Wu et al. 2022). The absence of double peaks in the SNPs' position that distinguish the subgenomes indicated a single *ALS* amplification.

**TABLE 1:** *ALS* primers utilized for partial *ALS* gene sequencing, copy number variation, global *ALS* relative expression, and specific *ALS* relative expression of subgenomes A, B, and C.

	Primer	Sequence (5′–3′)	Fragment size	Annealing T°C	References
Sequencing (Ala122, Pro197, Ala205, Phe206 positions)	EcALS6F	CGACGTCTTCGCCTACCC	538 bp	69–50 (touch‐down)	Turra et al. (2023 )
	EcALS5R	CACCTGCTCAAGCAATTCAG			
Sequencing (Trp574, Ser653, Gly654 positions)	EcALS2F	CATCATTGCCACTGGTGTTG	574 bp	60	Turra et al. (2023 )
	EcALS1R	ATACACGGTCCTGCCATCAC			
Copy number/global *ALS* expression	ALS_F	TGGCAGCTTCCTCATGAACAT	100 bp	60	
	ALS_R	ATCCCCAGGTGTTGGTTGTTT			
*ALS*‐SubA expression	F1_590Tm	GAGCACACACATACTTGGGGCAT	140 bp	60	Panozzo et al. (2021 )
	R1_621	GAGCATCTTCTTAATTGCTGCACGG			
*ALS*‐SubB expression	ALS‐B_F	AGTTGGCTATGATCCGCATC	113 bp	64	
	ALS‐B_R	TGCTCTGTTGGCCTTGTAGA			
*ALS*‐SubC expression	ALS‐C_F	GATATATCCGGATTTCGTGACC	111 bp	64	
	ALS‐C_R	CCTGGAGTCTCGAGCATCTT			

### 2.3 Dose–Response Curves to Four ALS Inhibitor Chemical Groups

Eight *E. crus‐galli* populations with different mutations from the previous experiment, and two susceptible populations, were selected to perform dose–response curves. These selected populations were one population carrying Ala122Thr (BAGE‐01), one with Ala205Asn (SANTPAT‐01), three with Trp574Leu (CAMAQ‐01, CAPV‐03, and SAOJER‐01), and three with Ser653Asn (ARRGR‐01, PALMS‐01, and 423). These populations had been self‐pollinated for four generations to ensure homozygosity, and these inbred lines are referred to as biotypes. The herbicides sprayed were imazethapyr, penoxsulam, bispyribac‐sodium, and nicosulfuron, utilizing the rates described previously as reference label rates. The doses sprayed are described in Table S1, and each treatment had five replicates. The experiment parameters and spray conditions were the same as described above. Dry biomass was assessed at 28 DAT. The data were submitted to analysis of variation (ANOVA), and when the interaction between factors was significant (*p* ≤ 0.05), the averages were fitted to the three‐parameter log‐logistic nonlinear regression model using the *drc v. 3.0‐1* package in R (Ritz et al. 2015) as follows: y = *d*/(1 + ((*x*/*e*)^*b*^)), where y is the dry biomass, *x* is the herbicide dose, *b* is the curve slope at the inflection point, *d* is the upper limit, and *e* is the inflection point, representing the dose that reduced 50% of the dry biomass (GR_50_ parameter). The latest was utilized to compare the resistance level of each biotype.

### 2.4 Copy Number Variation

The DNA of the eight resistant biotypes and one susceptible utilized in the dose–response curve experiment was extracted following the CTAB method with some adaptations (Doyle and Doyle 1987). The *ALS* copy number variation was assessed utilizing a primer pair that targets the three *ALS* genes of *E. crus‐galli* (Table 1), and the housekeeping gene utilized was *GAPDH* (*glyceraldehyde‐3‐phosphate dehydrogenase*) (Table S2). The master mix used was SsoAdvanced Universal SYBR Green Supermix (Bio‐Rad) following the manufacturer's protocol instructions, 98°C for 3 min and 40 cycles of 98°C for 15 s and “annealing temperature” for 30s, followed by one step of 65°C for 5 s and 95°C for 50s, utilizing the equipment CFX96 Real‐time System (Bio‐Rad). The copy number variation was calculated relative to the susceptible biotype following the method 2^−ΔΔCt^ (Dussault and Pouliot 2006). Each biotype had four biological replicates and three technical replicates. The data were compared according to the ANOVA, and when significant (*p* ≤ 0.05), the averages were compared according to Tukey 5%.

### 2.5 Dose–Response Curve With Metabolic Inhibitors

The biotypes CAMAQ‐01 and SAOJER‐01 carrying the Trp574Leu mutation showed very different resistance levels to ALS inhibitors. Dose–response curves to penoxsulam in combination with the CYP450 inhibitor malathion (Oliveira et al. 2018) or the glutathione‐S‐transferase (GST) inhibitor NBD‐Cl (4‐chloro‐7‐nitrobenzofurazan) (Cummins et al. 2013) were performed utilizing the two *E. crus‐galli* biotypes CAMAQ‐01 and SAOJER‐01 and one susceptible MOSTS‐01. The penoxsulam doses were 0, 20, 60, 180, 540, 1620, 4860, and 14,580 g ha^−1^ for CAMAQ‐01 but 0, 60, 180, 540, 1620, 4860, 14,580, and 43,740 g ha^−1^ for the SAOJER‐01 and 0, 0.93, 1.87, 3.75, 7.5, 15, 30, and 60 g ha^−1^ for the susceptible MOSTS‐01, plus adjuvant Veget'Oil 0.5% v/v. Malathion was sprayed 2 h before the herbicide at 1300 g ha^−1^, whereas NBD‐Cl was sprayed 48 h before at 270 g ha^−1^ diluted in a 1:1 water/acetone solution. The experiment procedures, conditions, and data analysis followed the same described previously.

### 2.6 *ALS* Gene Expression Across Subgenomes

Seven *E. crus‐galli* biotypes, one susceptible (MOSTS‐01), three with the *ALS* mutation Trp574Leu (CAMAQ‐01, CAPV‐03, and SAOJER‐01), and three with Ser653Asn (ARRGR‐01, PALMS‐01, and 423) had leaf tissue collected from untreated plants and 48 h after penoxsulam treatment at 72 g ha^−1^, plus adjuvant MSO (methylated seed oil) 0.5% v/v, and spray volume of 200 L ha^−1^. The plants were grown in a growth chamber (14‐h light/10‐h dark, temperature at 30°C day/25°C night), and were sprayed with an automated spray chamber. The tissue of the two youngest leaves was immediately frozen in liquid nitrogen after collection, and the RNA was extracted utilizing the Direct‐zol RNA Miniprep Plus kit (ZymoResearch). The cDNA synthesis was performed using the iScript cDNA Synthesis kit (Bio‐Rad). The expression of the *ALS* from each subgenome was assessed utilizing specific primers targeting SNPs that differentiate them (Figure S2A). The primer pairs targeting specifically the *ALS* from subgenomes B and C were designed utilizing the Primer3Plus software (https://www.bioinformatics.nl/cgi‐bin/primer3plus/primer3plus.cgi) (Table 1), whereas the primer pair targeting *ALS* from subgenome A was obtained from Panozzo et al. (2021) (Table 1). The specificity of our primers is shown by the absence of double peaks at the position that differentiates the subgenomes (Figure S2B). The primer pair targeting all three *ALS* genes was the same utilized for copy number variation. The housekeeping genes utilized were *eIF4B1* (e*ukaryotic translation initiation factor 4B1*), *GAPDH*, *18S* (*18S ribosomal RNA*), *28S* (2*8S ribosomal RNA*), and *RUB* (*rubisco*) (Table S2), which are common control genes utilized in herbicide qPCR studies (Chen et al. 2017; Duhoux and Délye 2013; Liu et al. 2019; Wrzesińska et al. 2016). The *eIF4B1* gene was identified as the most stable according to the stability analysis using the algorithm Normfinder in the RefFinder platform. The qPCR was performed following the protocol described for the “copy number variation,” with four biological and three technical replicates. The relative expression was calculated according to the method 2^−ΔΔCt^ (Dussault and Pouliot 2006), utilizing the *ALS* from subgenome A of each biotype as the reference. The data were compared according to ANOVA, and when significant (*p* ≤ 0.05), the averages were compared according to Tukey 5%. Based on the relative expression results, we estimated the percentage contribution of each *ALS* subgenome to the total amount of *ALS* transcripts.

## 3 Results

**FIGURE 1:**
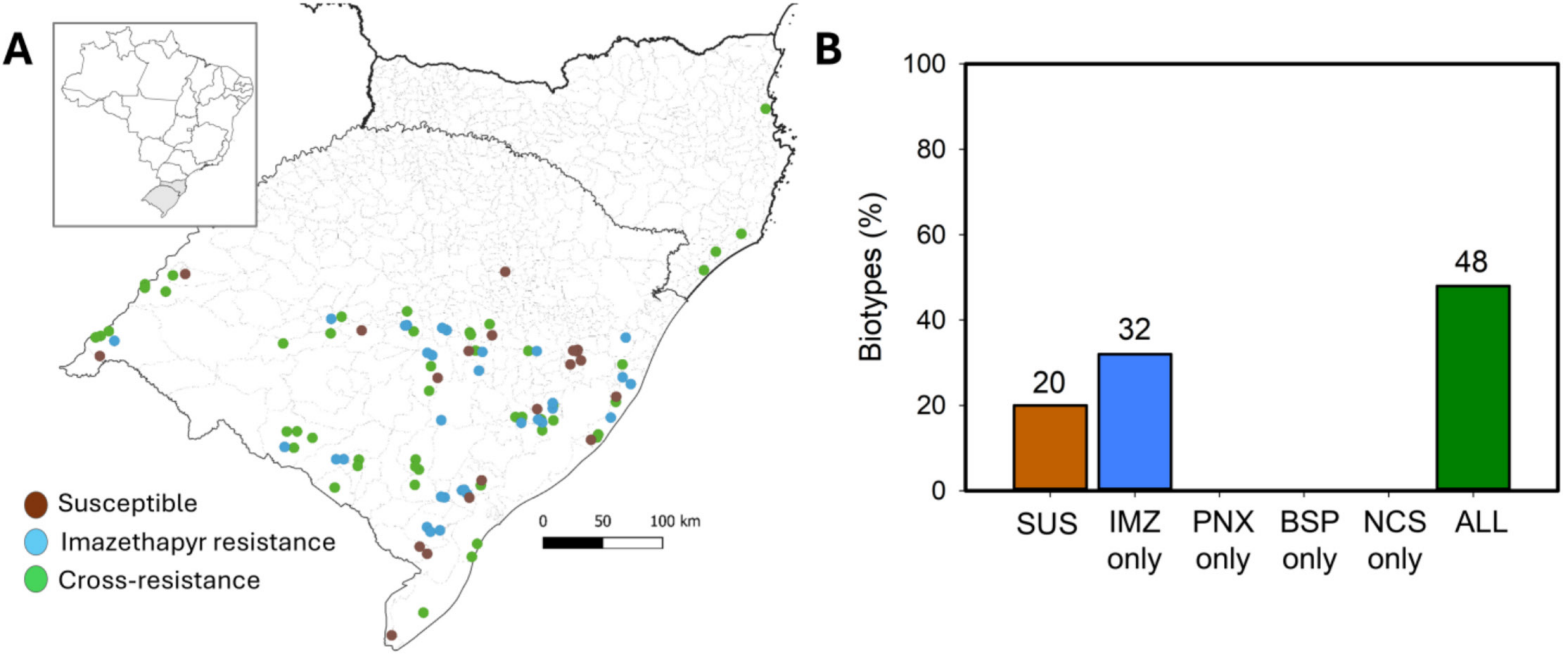
Mapping the resistance status of 100 *Echinochloa* biotypes in Southern Brazil and cross‐resistance characterization. (A) Distribution of *Echinochloa* biotypes susceptible and resistant to ALS inhibitor herbicides in Southern Brazil. (B) Percentage of biotypes characterized as susceptible (SUS), resistant to only one ALS inhibitor, or with cross‐resistance to all four ALS inhibitors tested: imazethapyr (IMZ), penoxsulam (PNX), bispyribac‐sodium (BSP), and nicosulfuron (NCS).

*E. crus‐galli* populations resistant to ALS inhibitors are spread across Southern Brazil, mainly in Rio Grande do Sul state (Figure 1A). Most of the populations evaluated, 48%, showed cross‐resistance to imazethapyr, penoxsulam, bispyribac‐sodium, and nicosulfuron, whereas 32% were resistant only to imazethapyr, and 20% were still susceptible to all ALS inhibitors tested (Figure 1B and Table S3). The western region of the state had more prevalence of cross‐resistant populations, whereas the eastern region had more imazethapyr single‐resistant and susceptible populations. All populations resistant to ALS inhibitors were resistant to imazethapyr (80%) (Figure 1B).

**FIGURE 2:**
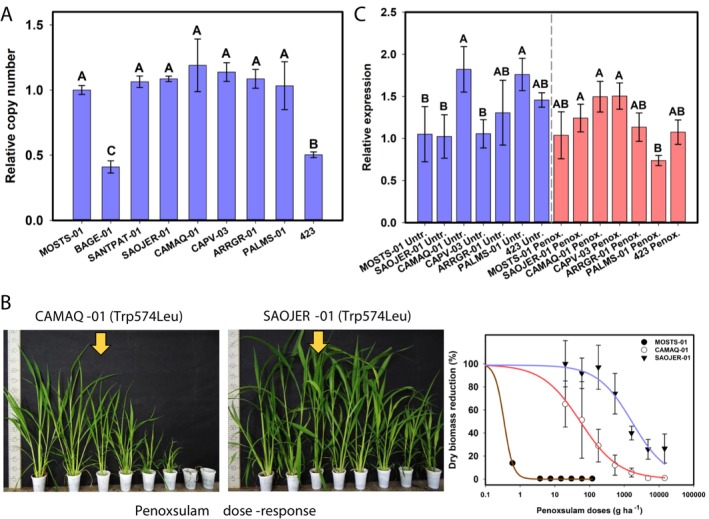
(A) *ALS* relative copy number analysis in *Echinochloa crus‐galli* biotypes showing different *ALS* mutations. (B) Differential resistance level to penoxsulam of two *E. crus‐galli* biotypes carrying the same Trp574Leu mutation in *ALS* gene but located in different subgenomes (CAMAQ‐01 in subgenome A; SAOJER‐01 in subgenome C). (C) Relative expression of total *ALS* (targeting the three subgenomes) in untreated plants (left) and 48 h after penoxsulam application (right). The yellow arrows indicate the label rate (60 g ha^−1^). Vertical error bars indicate the confidence interval (95% interval).

Four *ALS* mutations Ala122Thr, Ala205Asn, Trp574Leu, and Ser653Asn were identified in 18 *E. crus‐galli* populations evaluated (Table 2). We investigated the location of these *ALS* mutations in the allohexaploid *E. crus‐galli* genome. The *ALS* gene is located in Chr07 of subgenomes A, B, and C. Sixteen populations carrying Ala122Thr, Ala205Asn, Trp574Leu, or Ser653Asn had these mutations in *ALS* from subgenome A, whereas one biotype had a Trp574Leu in subgenome C, and one had a double mutation with Trp574Leu in subgenome A + Ala122Asn in subgenome C (Table 2). All the mutations conferred resistance to imazethapyr, but at different levels. The mutations Ala122Thr and Ala205Asn conferred intermediate imazethapyr resistance levels when compared to Trp574Leu (usually the highest) and Ser653Asn (usually the lowest) (Table 3). Penoxsulam, bispyribac‐sodium, and nicosulfuron resistance were observed only for biotypes carrying the Trp574Leu mutation (Table 3). Notably, biotypes with mutations Ala122Thr, Ala205Asn, and Ser653Asn also had higher RF to bispyribac‐sodium, nicosulfuron, and mainly penoxsulam when compared to the susceptible biotype; however, they were not considered resistant because they did not survive the herbicide label rates. No differences in *ALS* copy number that explain the herbicide resistance were observed when comparing the susceptible and resistant biotypes (Figure 2A). Two biotypes (BAGE‐01 and 423) showed half the number of *ALS* copies compared to the susceptible control, but they were not further investigated in this study.

**TABLE 2:** *ALS* mutations and their subgenome location in 18 *Echinochloa crus‐galli* biotypes resistant to ALS inhibitors from different counties in South Brazil.

Biotype	County	Subgenome A	Subgenome B	Subgenome C
MOSTS‐01 (susceptible)	Mostardas	—	—	—
BAGÉ‐01	Bagé	Ala122Thr	—	—
P47	Charqueadas	Ala122Thr	—	—
SANTPAT‐01	Sant. Ant. da Patrulha	Ala205Asn	—	—
CAMAQ‐01	Camaquã	Trp574Leu	—	—
CAPV‐01	Capivari do Sul	Trp574Leu	—	Ala122Asn
CAPV‐03	Capivari do Sul	Trp574Leu	—	—
MOSTS‐51	Mostardas	Trp574Leu	—	—
RIOGR‐01	Rio Grande	Trp574Leu	—	—
5.2 (RIOPAR)	Rio Pardo	Trp574Leu	—	—
386	Cachoeira do Sul	Trp574Leu	—	—
GBS‐46	Uruguaiana	Trp574Leu	—	—
424	Agudo	Trp574Leu	—	—
SAOJER‐01	São Jerônimo	—	—	Trp574Leu
ARRGR‐01	Arroio Grande	Ser653Asn	—	—
PALMS‐01	Palmares do Sul	Ser653Asn	—	—
423	Agudo	Ser653Asn	—	—
354	Arroio Grande	Ser653Asn	—	—
376	Arroio Grande	Ser653Asn	—	—

**TABLE 3:** Dose–response curve parameters to four ALS inhibitors (imazethapyr, penoxsulam, bispyribac‐sodium, and nicosulfuron) from different chemical groups (imidazolinones, triazolopyrimidine—type 2, pyrimidinylbenzoates, and sulfonylureas) utilizing *Echinochloa crus‐galli* biotypes with four different *ALS* mutations.

Biotypes	Mutation	*b*	*d*	*e* (GR_50_)	GR_50_ Lower limit	GR_50_ Upper limit	RF
		Imazethapyr					
MOSTS‐01	No	3.53*	4.23*	2.85*	2.19	3.51	—
CAPL‐01	No	3.69*	3.48*	2.80*	1.90	3.70	1.0
BAGE‐01	Ala122Thr	1.27*	4.78*	82.65*	60.20	105.11	29.0
SANTPAT‐01	Ala205Asn	1.55*	5.11*	92.60*	72.82	112.39	32.5
CAMAQ‐01	Trp574Leu	0.53*	3.06*	20.18^ns^	−11.52	51.89	7.1
CAPV‐03	Trp574Leu	0.50*	3.79*	134.31*	32.87	235.75	47.1
SAOJER‐01	Trp574Leu	0.58*	2.90*	449.87*	82.33	817.41	157.8
ARRGR‐01	Ser653Asn	1.54*	4.83*	43.72*	34.22	53.23	15.3
PALMS‐01	Ser653Asn	0.97*	6.27*	35.92*	27.22	44.62	12.6
423	Ser653Asn	2.14*	2.65*	279.05*	183.56	374.55	97.9
		Penoxsulam					
MOSTS‐01	No	0.93^ns^	5.98*	0.04^ns^	−0.14	0.23	—
CAPL‐01	No	1.11*	6.65*	0.15^ns^	−0.04	0.35	3.8
BAGE‐01	Ala122Thr	3.15*	3.15*	4.01*	3.02	5.01	100.3
SANTPAT‐01	Ala205Asn	0.75*	4.68*	9.50*	4.77	14.24	237.6
CAMAQ‐01	Trp574Leu	0.78*	3.27*	54.78*	12.50	97.05	1369.4
CAPV‐03	Trp574Leu	0.68*	3.12*	168.76*	34.27	303.25	4219.1
SAOJER‐01	Trp574Leu	0.79*	4.14*	1231.32*	597.30	1865.34	30783.0
ARRGR‐01	Ser653Asn	4.13^ns^	4.72*	2.40^ns^	−1.02	5.81	60.0
PALMS‐01	Ser653Asn	4.99^ns^	3.44*	2.82^ns^	−2.32	7.95	70.5
423	Ser653Asn	2.61^ns^	2.99*	2.29^ns^	−1.40	5.98	57.3
		Bispyribac‐sodium					
MOSTS‐01	No	1.89*	6.78*	0.63*	0.45	0.80	—
CAPL‐01	No	1.96*	3.92*	1.27*	0.87	1.67	2.0
BAGE‐01	Ala122Thr	3.97*	5.42*	3.51*	1.42	5.60	5.6
SANTPAT‐01	Ala205Asn	2.60*	5.89*	7.61*	6.55	8.67	12.1
CAMAQ‐01	Trp574Leu	0.77*	6.03*	20.60*	12.79	28.41	32.8
CAPV‐03	Trp574Leu	0.92*	2.91*	83.13*	43.96	122.31	132.2
SAOJER‐01	Trp574Leu	0.89*	5.43*	167.34*	98.95	235.72	266.1
ARRGR‐01	Ser653Asn	3.15*	4.62*	4.77*	3.84	5.70	7.6
PALMS‐01	Ser653Asn	3.44^ns^	6.15*	3.20^ns^	−4.08	10.48	5.1
423	Ser653Asn	1.53*	3.25*	4.99*	3.28	6.69	7.9
		Nicosulfuron					
MOSTS‐01	No	1.88*	6.01*	4.48*	3.42	5.54	—
CAPL‐01	No	3.67*	4.02*	12.11*	10.05	14.17	2.7
BAGE‐01	Ala122Thr	5.07*	4.60*	8.56*	6.71	10.42	1.9
SANTPAT‐01	Ala205Asn	2.54*	7.69*	17.38*	14.16	20.61	3.9
CAMAQ‐01	Trp574Leu	1.34*	6.79*	36.05*	27.04	45.07	8.1
CAPV‐03	Trp574Leu	1.55*	3.37*	81.23*	55.15	107.31	18.1
SAOJER‐01	Trp574Leu	1.28*	5.48*	155.99*	104.26	207.73	34.8
ARRGR‐01	Ser653Asn	5.78^ns^	4.58*	14.74*	13.31	16.17	3.3
PALMS‐01	Ser653Asn	10.16^ns^	4.77*	15.91*	9.17	22.66	3.6
423	Ser653Asn	3.36*	2.56*	24.51*	19.62	29.39	5.5

*Note:* GR_50_ = herbicide dose that reduced 50% of the dry biomass.

Abbreviations: ns = nonsignificant parameter, RF = resistance factor.

* Significant parameter (*p* < 0.05).

Resistance factor (RF) variation between biotypes carrying the same mutation was observed. The biotypes CAMAQ‐01 with Trp574Leu had the lowest RF to penoxsulam and imazethapyr, whereas SAOJER‐01, also with Trp574Leu, had the highest RF among all populations evaluated. The SAOJER‐01 was 3.3 times more resistant to imazethapyr than CAPV‐03 and 22.3 times more than CAMAQ‐01, all three with Trp574Leu (Table 3). The differential resistance levels between the three populations carrying Trp574Leu were observed for all four ALS inhibitors tested (Table 3). The dose–response curve showing the differential response to penoxsulam is exemplified in Figure 2B and Table S4. According to the sequencing, the biotypes CAMAQ‐01 (with the smallest RF) and CAPV‐03 have the Trp574Leu mutation in subgenome A, whereas SAOJER‐01 (with the highest RF) has the same mutation in subgenome C. A clear RF difference between biotypes carrying the same mutation Ser653Asn was also observed. The biotype 423 was 6.4 and 7.8 times more resistant to imazethapyr than ARRGR‐01 and PALMS‐01, respectively (Table 3). However, all three biotypes—ARRGR‐01, PALMS‐01, and 423—carry the Ser653Asn mutation in subgenome A (Table 2).

A metabolic inhibitor study was performed to understand the resistance level differences between the contrasting biotypes SAOJER‐01 and CAMAQ‐01 carrying Trp574Leu. No effect on resistance occurred with the GST inhibitor, but a strong CYP450 inhibitor effect was observed in both CAMAQ‐01 and SAOJER‐01 biotypes sprayed with penoxsulam, with an approximately 12‐fold GR_50_ reduction in CAMAQ‐01 and an approximately > 7‐fold GR_50_ reduction in SAOJER‐01 due to the CYP450 inhibitor application (Table 4). This result indicates that both biotypes have the CYP450 metabolic mechanism acting on herbicide detoxification in addition to the target site resistance mutation. The RF to penoxsulam (without inhibitor) for both CAMAQ‐01 and SAOJER‐01 in this second experiment (Table 4) was higher than the previous experiment (Table 3), indicative of an environmental effect on phenotypic resistance level that is common with metabolic resistance (Gaines et al. 2020). Note that the penoxsulam doses sprayed are different in both experiments and may result in slight variations in the estimation of dose–response parameters. However, SAOJER‐01 was still much more resistant to penoxsulam than CAMAQ‐01 when treated with the CYP450 inhibitor, which may suggest some additional mechanism contributing to the difference in resistance level between them.

**TABLE 4:** Penoxsulam dose–response curve investigating the presence of metabolic resistance in two resistant *Echinochloa crus‐galli* biotypes with the same Trp574Leu mutation but showing different resistance levels and one susceptible. Malathion and NBD‐Cl were the CP450 and GST enzyme metabolic inhibitors, respectively.

Biotypes	Inhibitors	*b*	*d*	*e* (*GR*_*50*_)	GR_50_ Lower limit	GR_50_ Upper limit	RF^a^
MOSTS‐01	No	5.34^ns^	3.87*	0.77^ns^	0.42	1.11	—
	CP450	3.80^ns^	4.17*	0.55^ns^	−0.22	1.32	0.71
	GST	4.71^ns^	3.60*	0.68*	0.04	1.32	0.88
CAMAQ‐01	No	0.47*	3.81*	132.94*	59.94	205.93	—
	CYP450	0.33*	3.16*	11.16^ns^	−4.01	26.33	0.08
	GST	0.34*	3.04*	140.07*	7.21	272.92	1.05
SAOJER‐01	No	0.35*	3.05*	> 43,740^ns^	—	—	—
	CYP450	0.26*	3.57*	6032.30*	447.11	11617.55	< 0.14
	GST	0.34*	2.98*	> 43,740^ns^	—	—	1.00

Abbreviation: ns = nonsignificant parameter.

^a^ Resistance factor (RF) compares the GR_50_ of metabolic inhibitors with that of no metabolic inhibitor in each biotype.

* Significant parameter (*p* < 0.05).

**FIGURE 3:**
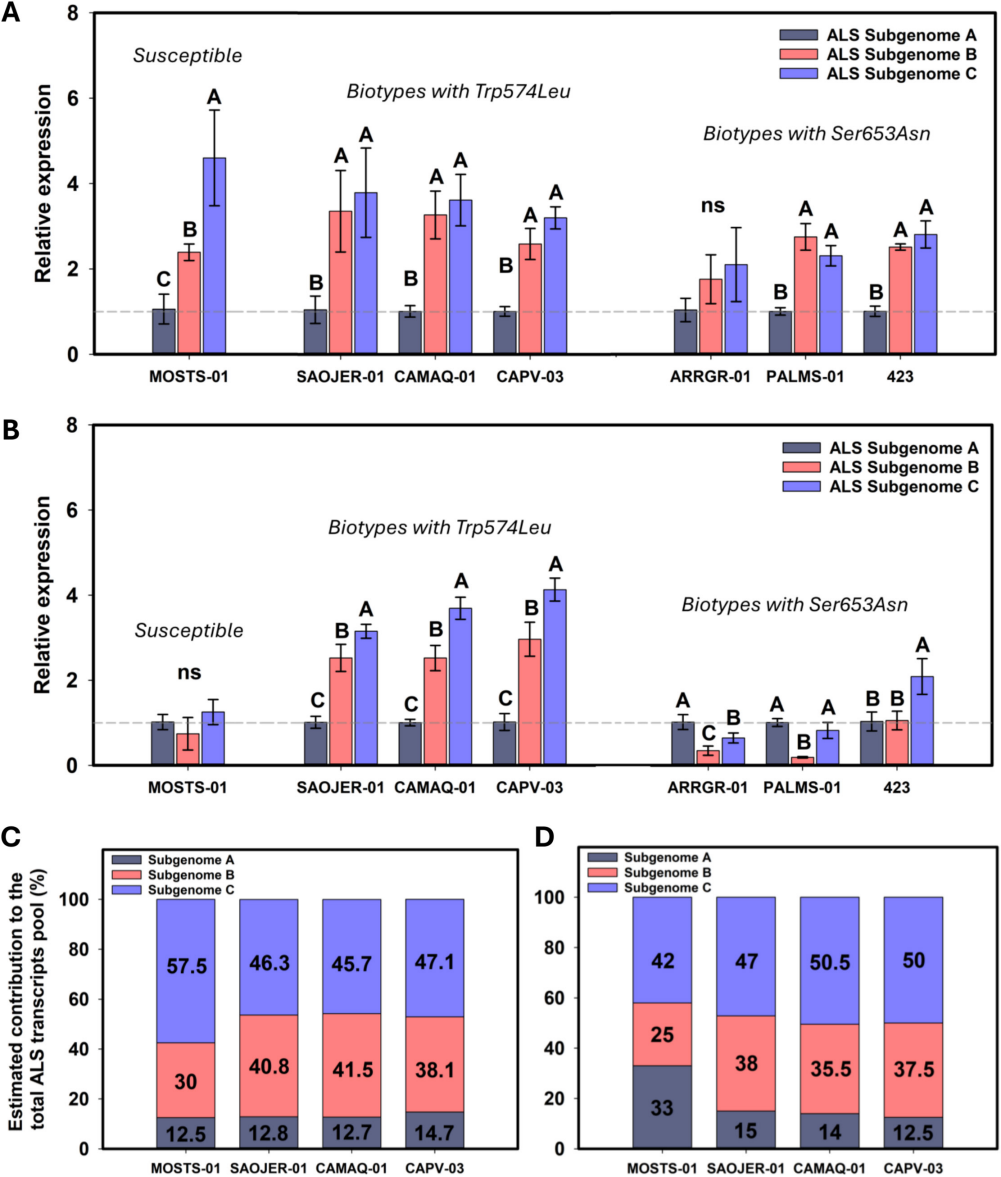
(A) Relative expression of *ALS*‐SubA, *ALS*‐SubB, and *ALS*‐SubC in one susceptible biotype, three with Trp574Leu mutation, and three with Ser653Asn mutation without herbicide application and (B) 48 h after penoxsulam application. (C) Estimated contribution based on relative expression analysis of each subgenome to the total *ALS* transcripts pool in one susceptible and three biotypes with Trp574Leu mutation without herbicide application and (D) 48 h after penoxsulam application. Capital letters compare the different subgenomes in the same biotype according to Tukey 5%. Vertical error bars indicate the confidence interval (95% interval).

No differences in global *ALS* expression (targeting all three subgenomes) were observed to explain the different RF levels between the susceptible and CAMAQ‐01, SAOJER‐01, CAPV‐03, ARRGR‐01, PALMS‐01, and 423 resistant biotypes (Figure 2C). However, differential *ALS* expression of each subgenome in each population was observed. All populations carrying Trp574Leu, Ser653Asn, and susceptible had higher expression of *ALS* from subgenomes C and B than A, except ARRGR‐01 in the absence of penoxsulam (Figure 3A). After spraying penoxsulam, the expression of *ALS* from subgenome C is the one with higher expression, whereas the *ALS* from subgenome A was the least expressed in penoxsulam‐resistant populations with Trp574Leu mutation (Figure 3B). The populations with Ser653Asn and susceptible showed a different pattern of *ALS* subgenome expression after penoxsulam treatment (Figure 3B). These biotypes were susceptible to penoxsulam, and the expression of *ALS* from each subgenome at 48 h after herbicide spray may be a consequence of cellular death. Utilizing the relative expression results, we estimated the contribution of *ALS* from each subgenome to the total amount of *ALS* transcripts (Figure 3C,D). In untreated samples, the *ALS* from subgenome C contributed approximately 46%–47% of the total *ALS* transcripts, whereas the *ALS* from subgenome A contributed about 13%–15% in populations with Trp574Leu mutation (Figure 3C). After penoxsulam spray, the *ALS* from subgenome C contributed 47%–50%, whereas subgenome A contributed 12%–15% (Figure 3D). SAOJER‐1 is the most herbicide‐resistant biotype and also the only biotype with a Trp574Leu mutation in subgenome C, which is the most expressed subgenome. The least resistant biotypes are those with the Trp574Leu mutation in subgenome A, which is the least expressed subgenome (Tables 2 and 3 and Figure 3A,B). The higher quantity of mutant transcripts in the total pool of *ALS* transcripts in SAOJER‐01 perhaps makes it more resistant. The highest penoxsulam dose tested in our experiment, 43,740 g ha^−1^ (which represents 729x the label rate), was not able to kill the SAOJER‐01 plants (Table 4).

## Discussion

The screening of 100 populations collected in Southern Brazil shows the distribution of ALS inhibitors resistance in *E. crus‐galli* . All resistant populations were resistant to imazethapyr; however, not all imazethapyr‐resistant populations were resistant to penoxsulam, bispyribac‐sodium, or nicosulfuron, considering the recommended label rate. In addition, when the population showed resistance to one of the herbicides penoxsulam, bispyribac‐sodium, or nicosulfuron, it was also resistant to the other two. Four *ALS* mutations were characterized in association with the different patterns of cross‐resistance in *E. crus‐galli* . The populations surviving imazethapyr, penoxsulam, bispyribac‐sodium, and nicosulfuron application had the *ALS* mutation Trp574Leu. The populations surviving only imazethapyr had one of the three following mutations: Ala122Thr, Ala205Asn, or Ser653Asn. These mutated *ALS* positions identified in our study have already been confirmed to confer herbicide resistance through ALS enzymatic activity assays in other studies (Brosnan et al. 2016; Deng et al. 2017; Cao et al. 2022; Palmieri et al. 2022).

Different than the cross‐resistance pattern we identified in our study, in *Bromus tectorum* , the Ser653Asn mutation, other than imidazolinone chemical group resistance, also conferred resistance to pyroxsulam, which belongs to the same chemical group as penoxsulam (triazolopyrimidine), however still susceptible to the sulfonylurea chemical group (Kumar and Jha 2017); in *Setaria viridis* , the same mutation conferred resistance to imidazolinone and also to sulfonylureas (Laplante et al. 2009). In *E. crus‐galli* biotypes from our study, we could link cross‐resistance to each mutation, but not resistance level, because different biotypes carrying the same mutation Trp574Leu or Ser653Asn had different RF. Besides reporting the *ALS* mutations in *E. crus‐galli* and the different cross‐resistance patterns, we identified that they are prevalent in subgenome A in *E. crus‐galli* . Only the biotypes SAOJER‐01 and CAPV‐01 had a mutation in *ALS* from subgenome C, Trp574Leu and Ala122Asn, respectively. The occurrence of mutations in all three *E. crus‐galli* *ALS* subgenomes has been reported (Löbmann et al. 2021; Yang et al. 2021). However, our study is the first to sequence multiple hexaploid biotypes and evaluate in which subgenome *ALS* mutations are prevalent.

The fact that all four mutations identified confer resistance to imazethapyr, whereas only one to penoxsulam, bispyribac‐sodium, and nicosulfuron, correlates with the adoption of rice cultivars resistant to imidazolinone herbicides (Clearfield rice) that have been cultivated in Brazil since the mid‐2000s (Avila et al. 2021). The recurrent use of imazethapyr in Clearfield rice for about 20 years has selected *E. crus‐galli* *ALS* mutations to survive imazethapyr. The use of penoxsulam and bispyribac‐sodium before and concomitantly with imazethapyr in Clearfield rice also selects mutations associated with these herbicides and imazethapyr. Penoxsulam and bispyribac‐sodium use has been reduced since the availability of Clearfield rice because they are not effective in controlling weedy rice. Our results show that penoxsulam and bispyribac‐sodium are still effective in controlling 52% of the Ec biotypes evaluated (32% resistant only to imazethapyr + 20% susceptible), which highlights that some ALS inhibitor active ingredients are still valuable to control *E. crus‐galli* . Research papers and scientific communication sometimes generalize the resistance to one active ingredient as resistance to “ALS inhibitors,” which is not always true and indirectly discourages the applications of the other ALS inhibitor active ingredients. In a scenario where *Echinochloa* spp. have been evolving resistance to most of the rice selective herbicides, and few or no options are still effective in several rice fields, the understanding of *ALS* mutation cross‐resistance patterns is important to design a rational ALS chemical group rotation.

There are several studies in *E. crus‐galli* reporting *ALS* mutations and cross‐resistance patterns, but the continuous understanding of cross‐resistance patterns is important because they may change in different populations of the same weed species. For example, the mutation Ala122Thr reduced the ALS enzyme sensitivity to imazamox and penoxsulam in a previous study (Riar et al. 2012, 2013), whereas in our results, the population carrying the same mutation showed resistance only to imazethapyr. The complexity of cross‐resistance patterns observed in *E. crus‐galli* and other species supports the continuous necessity of studies diving into the target site resistance mechanism. In addition, different levels of resistance in biotypes carrying the same mutation were observed. The biotype SAOJER‐01 was 22 times more resistant to imazethapyr and penoxsulam than CAMAQ‐01; when comparing SAOJER‐01 to the biotype CAPV‐03, it was three and seven times more resistant to imazethapyr and penoxsulam, respectively. All three carry the Trp574Leu mutation in the *ALS* gene. The biotype 423 was at least 6.5 times more resistant to imazethapyr than ARRGR‐01 and PALMS‐01, all three carrying the Ser653Asn mutation. These variabilities may be explained by different genetic backgrounds, such as differential capacity to metabolize the herbicides. Very often, target site and nontarget site herbicide resistance mechanisms occur simultaneously in the same plant (Kaspary et al. 2025; McElroy and Hall 2020). To investigate it, we studied the two most contrasting biotypes, SAOJER‐01 and CAMAQ‐01. We identified that CYP450‐mediated penoxsulam metabolism was present in both SAOJER‐01 and CAMAQ‐01 biotypes, due to the CYP450 inhibitor effect. Metabolic resistance in SAOJER‐01 was also reported for cyhalofop‐butyl (Cutti et al. 2026). Several *CYP* genes have been characterized to metabolize penoxsulam in other *Echinochloa* sp. studies, such as *CYP81A12*, *CYP81A21* (Iwakami et al. 2014), and *CYP72A15* (Zhang et al. 2025) in *E. phyllopogon* and *CYP81A68* in *E. crus‐galli* (Pan et al. 2022). The GST inhibitor did not show any effect in reducing the resistance to penoxsulam in our study, but some *GST* genes have also been identified as metabolizing it, such as *GST1* in *E. phyllopogon* (Gao et al. 2025) and *GST4* in *E. crus‐galli* (Fang et al. 2025). The resistance level difference between SAOJER‐01 and CAMAQ‐01 was still high, even after metabolic inhibitor application. We cannot discard the occurrence of other metabolic mechanisms in SAOJER‐01 that were not inhibited by the metabolic inhibitors tested.

When comparing the biotypes carrying the Trp574Leu mutation, the CAMAQ‐01 and CAPV‐03 have it in subgenome A and showed lower resistance than SAOJER‐01, which has it in subgenome C. We further investigated the expression level of *ALS* from each subgenome in six resistant biotypes and one susceptible. The *ALS* genes from subgenomes B and C were most expressed, whereas the one in subgenome A was the least expressed in all biotypes analyzed, except in ARRGR‐01. The upregulation of *ALS* from subgenome C with the Trp574Leu mutation increases the dosage of the resistant allele, increasing the number of mutated transcripts in the mRNA pool. This is probably a case of unbalanced expression resulting in a differential dilution effect of mutant enzymes reflected in the resistance level. Another study shows *E. oryzicola* (tetraploid and genitor of *E. crus‐galli* ) with the *ALS* mutation Trp574Leu in *ALS1* (the *ALS* from subgenome A) (Panozzo et al. 2021), which corroborates our results, where most mutations were also in subgenome A. However, their findings show higher expression of *ALS1* from subgenome A than from subgenome B. Corroborating our results, the analysis of the differential expression of *E. crus‐galli* subgenomes revealed that a significantly higher proportion of suppressed genes occurred in subgenome A than in subgenomes B and C (Ye et al. 2020). On average, 14% of homologous genes in *E. crus‐galli* showed single‐homologous dominance, whereas 30.6% exhibited single‐homologous suppression (Ye et al. 2020). The subgenome dominance may be driven by the presence of transposable elements, which can cause changes in the pattern of gene expression through *cis*‐regulation of nearby genes or *trans*‐regulation producing short interfering RNAs (Gill et al. 2021). Dominant subgenomes have significantly lower transposable element density compared to the submissive subgenome (Alger and Edger 2020). In general, the presence of herbicide target site mutations in one subgenome is sufficient to confer a herbicide‐resistant phenotype, and it does not matter whether that subgenome has higher or lower expression than the others.

Differential herbicide resistance levels according to the subgenome location of the mutation have been reported in a few polyploid species. In wheat ( *Triticum aestivum* ), the *ALS* Ser653Asn mutation in subgenome D conferred higher resistance to imazethapyr than in subgenome A or B (Chen et al. 2021). Also in wheat, the *ACCase* Ala2004Val mutation in subgenome A or D conferred higher resistance to quizalofop‐p‐ethyl than in subgenome B (Ostlie et al. 2015). However, in tetraploid *Sonchus oleraceus* , the presence of Pro197Ser in *ALS* from different subgenomes did not confer differential resistance to metsulfuron‐methyl or imazamox + imazethapyr (Merriam et al. 2023). Different species with different ploidy levels likely have different genetic regulation mechanisms, so the results from one species cannot be directly translated to another.

The resistance level or cross‐resistance pattern of mutations is influenced by the key factors. (1) *Location of the mutation*: Amino acid replacements in different locations in the gene sequence may result in resistance to different herbicides from the same mode of action; for example, Trp574Leu confers resistance to all ALS inhibitors, whereas mutations in other positions provide resistance to only one or more herbicides, as shown in our study. (2) *Amino acid replacement*: Different amino acids in the same position may change the affinity to different herbicides, such as Ala122Asn showing cross‐resistance to four ALS inhibitors, but our Ala122Thr only to one, and our Trp574Leu showed resistance to the imidazolinone chemical group, whereas Trp574Arg showed sensitivity to imidazolinones (Fang et al. 2019; Panozzo et al. 2017). (3) *Ploidy level and number of alleles mutated*: The presence of a mutation in one homologous gene in a polyploid has a dilution effect in the pool of total enzymes reducing the resistance level in polyploids when compared to diploids (Yu et al. 2013). For example, the mutation Ser653Asn located only in subgenome A conferred a low‐resistance level in our hexaploid *E. crus‐galli* biotypes but showed stronger resistance in diploid weedy rice (Ruzmi et al. 2020), *Amaranthus tuberculatus* (Patzoldt and Tranel 2007), and *A. hybridus* (Whaley et al. 2006). In addition, the presence of the Ser653Asn mutation in both B and D subgenomes of wheat conferred higher imazamox resistance than the mutation in only B or D subgenome (Hanson et al. 2006). In the same way, diploids with more than one copy of the herbicide target encoding gene in the genome show higher resistance when all genes carry a mutation; for example, soybean carrying the Pro197Ser and Trp574Leu in *ALS1* and *ALS2*, respectively, showed higher ALS inhibitors resistance than the isolated mutations (Walter et al. 2014). Homozygosity or heterozygosity works in the same way. (4) *Species/population*: Different species or populations of the same species have different innate or selected increased ability to detoxify herbicides before they reach the target site, which could explain why a mutation confers different resistance patterns or resistance levels than expected. For example, the *E. crus‐galli* *ALS* mutation Ala122Thr reduced the enzyme sensitivity to penoxsulam (Riar et al. 2013), but the population with the same mutation in our study was susceptible to penoxsulam. The findings in our study and from others (Chen et al. 2021; Ostlie et al. 2015) allow us to add one more factor that does not affect the cross‐resistance pattern but strongly affects the resistance level. (5) *Subgenome location of the mutation*: The location of the mutated gene in different polyploid subgenomes may result in different contributions to the total pool of transcripts, affecting the resistance level.

Knowledge of herbicide resistance in polyploid species has recently expanded with the generation of genomic resources, unraveling the sequence of multiple herbicide target‐encoding genes. We identified that *ALS* mutations Trp574Leu, Ala122Thr, Ala205Asn, and Ser653Asn in *E. crus‐galli* are prevalent in subgenome A, the lowest expressed in the biotypes evaluated, whereas two biotypes showed a mutation in *ALS* from subgenome C, which was the highest expressed. Biotypes with the same *ALS* mutation exhibited different resistance levels. One biotype with Trp574Leu mutation in subgenome C was more resistant to all ALS inhibitors than the two other biotypes with the same mutation but in subgenome A. In addition to the Trp574Leu, the CYP450‐mediated metabolism also contributes to penoxsulam resistance. All four mutations identified conferred resistance to imazethapyr, but only Trp574Leu also conferred resistance to penoxsulam, bispyribac‐sodium, and nicosulfuron, when considered the herbicide label rates. The rotation of ALS inhibitor chemical groups could be a strategy to control the imidazoline‐resistant populations carrying Ala122Thr, Ala205Asn, and Ser653Asn mutations.

## Author Contributions

Conceptualization: L.C. and A.M. Methodology: L.C., G.M.T., P.S.A., E.S., F.M.M., C.M., T.A.G., and A.M. Investigation: L.C., A.M., and T.A.G. Funding acquisition: A.M. and T.A.G. Project administration: L.C., A.M., and T.A.G. Supervision: A.M. and T.A.G. Writing – original draft: L.C., A.M., and T.A.G. Writing – review and editing: L.C., A.M., T.A.G., C.M., G.M.T., P.S.A., E.S., and F.M.M.

## Funding

The authors have nothing to report.

## Conflicts of Interest

The authors declare no conflicts of interest.

## Supporting information


**Figure S1:** Alignment of the whole *ALS* gene from three subgenomes showing the SNPs that differentiate them and specific primers targeting each one. The sequences are from Genbank: MH013494.1 (*ALS*‐SubA), MG188321.1 (*ALS*‐SubB), and MH013493.1 (*ALS*‐SubC). Asterisk (*) indicates the same nucleotide in all three subgenomes. Absence of * indicates there is at least one different nucleotide between them.
**Figure S2:** Confirmation of specificity of the primers targeting the three *ALS* from different subgenomes in expression analysis. (A) Partial *Echinochloa crus‐galli* *ALS* sequence showing the SNPs that differentiate them in each subgenome, and the forward primers targeting the SNPs at the 3′‐end. (B) Sequencing of the PCR product when utilizing the *ALS*‐specific primers targeting subgenome B or C, showing the absence of a double peak at the primer 3′‐end where the specific SNPs are.
**Table S1:** Doses of four ALS inhibitors utilized for the dose–response curve with *Echinochloa crus‐galli* biotypes with different *ALS* mutations.
**Table S2:** Primers utilized as reference in the copy number variation and relative gene expression assays.
**Table S3:** Screening of 100 *Echinochloa* populations from Southern Brazil with four ALS‐inhibitor chemical groups: imazethapyr, penoxsulam, bispyribac‐sodium, and nicosulfuron from imidazolinones, triazolopyrimidine—type 2, pyrimidinylbenzoates, and sulfonylureas, respectively. Efficacy of control 28 days after treatment (0% = no injuries; 100% = dead).
**Table S4:** Dose–response curve to penoxsulam utilizing biomass (g) and herbicide efficacy (%) variables of two *Echinochloa crus‐galli* biotypes with Trp574 mutation in *ALS* gene (CAMAQ‐01 and SAOJER‐01) and one susceptible without mutation (MOSTS‐01).

## Data Availability

All the data are available in the manuscript or in the Supporting Information. Additional data will be made available upon request.
