# Supplementary material for: Subgenomic Distribution and Herbicide Cross‐Resistance of ALS Gene Mutations in Allohexaploid Echinochloa crus‐galli
Source: Plant Direct. 2026 Apr 21;10:e70168. doi: 10.1002/pld3.70168 (PMC13099262; doi:10.1002/pld3.70168)
Supplement: Supplementary file 1 — Figure S1: Alignment of the whole ALS gene from three subgenomes showing the SNPs that differentiate them and specific primers targeting each one. The sequences are from Genbank: MH013494.1 (ALS‐SubA), MG188321.1 (ALS‐SubB), and MH013493.1 (ALS‐SubC). Asterisk (*) indicates the same nucleotide in all three subgenomes. Absence of * indicates there is at least one different nucleotide between them. Figure S2: Confirmation of specificity of the primers targeting the three ALS from different subgenomes in expression analysis. (A) Partial Echinochloa crus‐galli ALS sequence showing the SNPs that differentiate them in each subgenome, and the forward primers targeting the SNPs at the 3′‐end. (B) Sequencing of the PCR product when utilizing the ALS‐specific primers targeting subgenome B or C, showing the absence of a double peak at the primer 3′‐end where the specific SNPs are. Table S1: Doses of four ALS inhibitors utilized for the dose–response curve with Echinochloa crus‐galli biotypes with different ALS mutations. Table S2: Primers utilized as reference in the copy number variation and relative gene expression assays. Table S3: Screening of 100 Echinochloa populations from Southern Brazil with four ALS‐inhibitor chemical groups: imazethapyr, penoxsulam, bispyribac‐sodium, and nicosulfuron from imidazolinones, triazolopyrimidine—type 2, pyrimidinylbenzoates, and sulfonylureas, respectively. Efficacy of control 28 days after treatment (0% = no injuries; 100% = dead). Table S4: Dose–response curve to penoxsulam utilizing biomass (g) and herbicide efficacy (%) variables of two Echinochloa crus‐galli biotypes with Trp574 mutation in ALS gene (CAMAQ‐01 and SAOJER‐01) and one susceptible without mutation (MOSTS‐01). [file PLD3-10-e70168-s001.docx]

**SUPPLEMENTARY MATERIAL**

**Subgenomic distribution and herbicide cross-resistance of *ALS* gene mutations in hexaploid *Echinochloa crus-galli***

Short title: Subgenomic *ALS* mutations and herbicide resistance in barnyardgrass

Luan Cutti^a^, Guilherme Menegol Turra^b^, Filipi Mesquita Machado^c^, Estéfani Sulzbach^d^, Paula Sinigaglia Angonese^e^, Catarine Markus^f^, Todd A. Gaines^g*^, Aldo Merotto^h*^

^a^ Crop Science Department, Federal University of Rio Grande do Sul, Porto Alegre, RS, Brazil. ORCID: 0000-0002-2867-7158. E-mail: luancutti@hotmail.com

^b^ Crop Science Department, Federal University of Rio Grande do Sul, Porto Alegre, RS, Brazil. ORCID: 0000-0002-4806-7613. E-mail: turragm@gmail.com

^c^ Crop Science Department, Federal University of Rio Grande do Sul, Porto Alegre, RS, Brazil. ORCID: 0000-0003-2113-0907. E-mail: filipimesqmach@gmail.com

^d^ Crop Science Department, Federal University of Rio Grande do Sul, Porto Alegre, RS, Brazil. ORCID: 0000-0002-8699-6640. E-mail: estefanisulzbach@gmail.com

^e^ Crop Science Department, Federal University of Rio Grande do Sul, Porto Alegre, RS, Brazil. ORCID: 0000-0001-7947-8178. E-mail: paulasangonese@gmail.com

^f^ Crop Science Department, Federal University of Rio Grande do Sul, Porto Alegre, RS, Brazil. ORCID: 0000-0002-5330-3502. E-mail: catarine.markus@ufrgs.br

^g^ Department of Agricultural Biology, Colorado State University, Fort Collins, Colorado, USA. ORCID: 0000-0003-1485-7665. E-mail: todd.gaines@colostate.edu

^h^ Crop Science Department, Federal University of Rio Grande do Sul, Porto Alegre, RS, Brazil. ORCID: 0000-0002-1581-0669. E-mail: aldo.merotto@ufrgs.br

*Corresponding authors

ALS-A ATGGCCACGACCGCCGCCGCCACCGCCGCCACGGCGGCCGCCGCGCTCACCGGCGCCACC 60

ALS-B ATGGCCACGACCGCCGCCGCCACCGCCGCCACGGCGGCCGCCGCGCTCACCGGCGCCACC 60

ALS-C ATGGCCACGACCGCCGCCGCCACCGCCGCCACGGCGGCCGCCGCGCTCACCGGCGCCACC 60

************************************************************

ALS-A ACCGCCGCGCCCAGGCCGAGCCGCCACGGTTACTCCGCGGCGGCCGCCCGCCGCGCCGCG 120

ALS-B ACCGCCGCGCCCAGGCCGAGCCGCCACGGTTACTCCGCGGCGGCCGCCCGCCGCGCCGCG 120

ALS-C ACCGCCGCGCCCAGGCCGAGCCGCCACGGTTACTCCGCGGCGGCCGCCCGCCGCGCCGCG 120

************************************************************

ALS-A CCCATCCGGTGCTCCGCGGCGTCGCCGGCCACGGCCACGGCTCCCCCGGCCACCCCGCTC 180

ALS-B CCCATCCGGTGCTCCGCGGCGTCGCCGGCCACGGCCACGGCTCCCCCGGCCACCCCGCTC 180

ALS-C CCCATCCGGTGCTCCGCGGCGTCGCCGGCCACGGCCACGGCTCCCCCGGCCACCCCGCTC 180

************************************************************

ALS-A CGACCGTGGGGCCCCACCGAGCCGCGCAAGGGCGCCGACATCCTCGTCGAGGCCCTCGAG 240

ALS-B CGACCGTGGGGCCCCACCGAGCCGCGCAAGGGCGCCGACATCCTCGTCAAGGCCCTCGAG 240

ALS-C CGACCGTGGGGCCCCACCGAGCCGCGCAAGGGCGCCGACATCCTCGTCGAGGCCCTCGAG 240

************************************************ ***********

ALS-A CGCTGCGGCGTCCGCGACATCTTCGCCTACCCCGGCGGCGCCTCCATGGAGATCCACCAG 300

ALS-B CGCTGCGGCGTCCGCGACGTCTTCGCCTACCCCGGCGGCGCCTCCATGGAGATCCACCAG 300

ALS-C CGCTGCGGCGTCCGCGACATCTTCGCCTACCCCGGCGGCGCCTCCATGGAGATCCACCAG 300

****************** *****************************************

ALS-A GCGCTCACCCGCTCCCCCGTCATCGCCAACCACCTCTTCCGCCACGAGCAAGGGGAGGCC 360

ALS-B GCGCTCACCCGCTCCCCCGTCATCGCCAACCACCTCTTCCGCCACGAGCAAGGGGAGGCC 360

ALS-C GCGCTCACCCGCTCCCCCGTCATCGCCAACCACCTCTTCCGCCACGAGCAGGGGGAGGCC 360

************************************************** *********

ALS-A TTCGCCGCCTCCGGGTTCGCGCGCTCGTCCGGCCGCGTCGGCGTCTGCGTCGCCACCTCG 420

ALS-B TTCGCCGCCTCCGGGTTCGCGCGCTCGTCCGGCCGCGTCGGCGTCTGCGTCGCCACCTCG 420

ALS-C TTCGCCGCCTCCGGCTTCGCGCGCTCGTCCGGCCGCGTCGGCGTCTGCGTCGCCACCTCG 420

************** *********************************************

ALS-A GGCCCCGGCGCCACCAACCTCGTCTCCGCGCTCGCCGACGCGCTGCTCGACTCCATCCCC 480

ALS-B GGCCCCGGCGCCACCAACCTCGTCTCCGCGCTCGCCGACGCGCTGCTCGACTCCATCCCC 480

ALS-C GGCCCCGGCGCCACCAACCTCGTCTCCGCGCTCGCCGACGCGGTGCTCGACTCCATCCCC 480

****************************************** *****************

ALS-A ATGGTCGCCATCACCGGCCAGGTGCCCCGCCGCATGATCGGCACCGACGCCCTCCAGGAG 540

ALS-B ATGGTCGCCATCACCGGCCAGGTGCCCCGCCGCATGATCGGCACCGACGGCTTCCAGGAG 540

ALS-C ATGGTCGCCATCACCGGCCAGGTGCCCCGCCGCATGATCGGCACCGACGCCTTCCAGGAG 540

************************************************* * ********

ALS-A ACGCCAATCGTCGAGGTCACCCGCTCAATCACCAAGCACAACTACCTCGTCCTCGACATC 600

ALS-B ACGCCAATCGTCGAGGTCACCCGCCCCATCACCAAGCACAACTACCTCGTCCTCGACATC 600

ALS-C ACGCCAATCGTCGAGGTCACCCGCTCCATCACCAAGCACAACTACCTCGTCCTCGACATC 600

************************ * *********************************

ALS-A GACGACATCCCCCGCGTCGTGCAGGAGGCGTTCTTCCTCGCCTCCTCTGGCCGACCGGGG 660

ALS-B GACGACATCCCCCGCGTCATACAGGAGGCCTTCTTCCTCGCCTCCTCTGGCCGGCCCGGG 660

ALS-C GACGACATCCCCCGCGTCATACAGGAGGCGTTCTTCCTCGCCTCCTCTGGCCGGCCGGGG 660

****************** * ******** *********************** ** ***

ALS-A CCGGTGCTCGTCGACATCCCCAAGGACATCCAGCAGCAGATGGCCGTGCCGGTCTGGAAC 720

ALS-B CCGGTGCTCGTCGACATCCCCAAGGACATCCAGCAGCAGATGGCCGTGCCGGTCTGGAAC 720

ALS-C CCGGTGCTCGTCGACATCCCCAAGGACATCCAGCAGCAGATGGCCGTGCCCGTCTGGAAC 720

************************************************** *********

ALS-A ACGCCCATGAGTCTGCCGGGGTACATTGCGCGCCTGCCCAAGCCTCCGGCAACTGAATTG 780

ALS-B ACGCCCATGAGTCTGCCGGGGTACATTGCGCGCCTGCCCAAGCATCCGGCAACTGAATTG 780

ALS-C ACGCCCATGAGTCTGCCGGGGTACATTGCGCGCCTGCCCAAGCCTCCGGCAACTGAATTG 780

******************************************* ****************

ALS-A CTTGAGCAGGTGCTGCGTCTTGTTGGTGAGTCACGGCGCCCTGTTCTTTATGTTGGTGGT 840

ALS-B CTTGAGCAGGTGCTGCGTCTTGTTGGTGAGTCGCGGCGCCCTGTTCTTTATGTTGGTGGT 840

ALS-C CTTGAGCAGGTGCTGCGTCTTGTTGGTGAGTCACGGCGCCCTGTTCTTTATGTTGGTGGT 840

******************************** ***************************

ALS-A GGCTGCGCTGCATCCGGTGAGGAGCTGTGCCGCTTTGTGGAGATGACCGGAATCCCAGTG 900

ALS-B GGTTGCGCTGCATCCGGTGAGGAGCTGCGCCGCTTTGTGGAGATGACCGGAATCCCAGTG 900

ALS-C GGTTGCGCTGCATCCGGTGAGGAGCTGCGCCGCTTTGTGGAGATGACCGGAATCCCAGTG 900

** ************************ ********************************

ALS-A ACAACTACTCTGATGGGCCTTGGCAACTTCCCCAGTGATGACCCACTGTCTCTGCGCATG 960

ALS-B ACAACTACTCTAATGGGCCTTGGTAACTTCCCCAGTGATGATCCACTGTCTCTGCGCATG 960

ALS-C ACAACTACTCTGATGGGCCTTGGCAACTTCCCCAGTGATGACCCACTGTCTCTGCGCATG 960

*********** *********** ***************** ******************

ALS-A CTTGGTATGCACGGTACTGTATATGCAAATTATGCAGTGGATAAGGCCGACCTGTTGCTG 1020

ALS-B CTTGGTATGCACGGTACTGTATATGCAAATTATGCAGTGGATAAGGCCGACCTGTTGCTG 1020

ALS-C CTCGGTATGCACGGTACTGTATATGCAAATTATGCAGTGGATAAGGCCGACCTGTTGCTG 1020

** *********************************************************

ALS-A GCGTTTGGTGTGCGGTTCGATGATCGCGTGACAGGAAAAATTGAGGCTTTTGCAAGCAGG 1080

ALS-B GCATTTGGTGTGCGGCTCGATGATCGTGTGACAGGGAAAATTGAGGCTTTTGCAAGCAGG 1080

ALS-C GCATTTGGTGTGCGGTTCGATGATCGTGTGACAGGGAAAATTGGGGCTTTTGCAGGCAGG 1080

** ************ ********** ******** ******* ********** *****

ALS-A GCCAAGATTGTGCACATTGATATTGATCCAGCTGAGATTGGCAAGAACAAGCAGCCACAT 1140

ALS-B GCCAAGATTGTGCACATTGATATTGATCCAGCTGAGATTGGCAAGAACAAGCAGCCACAT 1140

ALS-C GCCAAGATTGTGCACATTGATATTGATCCAGCTGAGATTGGCAAGAACAAGCAGCCACAT 1140

************************************************************

ALS-A GTGTCCATCTGTGCGGATGTCAAGCTTGCTTTGCAGGGCATGAATGCTCTTCTGGAAGGA 1200

ALS-B GTGTCCATCTGTGCGGATGTCAAGCTTGCTTTGCAGGGCATGAATGCTCTTCTGGAAGGA 1200

ALS-C GTGTCCATCTGTGCGGATGTCAAGCTTGCTTTGCAGGGCATGAATGCTCTTCTGGAAGGA 1200

************************************************************

ALS-A ATCATATCAAAGAAGAGTTTTGACTTTGGCTCATGGCACGATGAGTTGGATCAGCAGAAG 1260

ALS-B ATCATATCAAAGAAGAGTTTTGACTTTGGCTCATGGCAAGATGAGTTGGATCAGCAGAAG 1260

ALS-C ATCATATCAAAGAAGAGTTTTGACTTTGGCTCATGGCAGGATGAGTTGGATCAGCAGAAG 1260

************************************** *********************

ALS-A AGGGAATTCCCCCTGGGGTACAAAACTTTCGATGAGGAGATTCAGCCACAGTATGCTATC 1320

ALS-B AGGGAATTCCCCCTGGGGTACAAAACTTTCGATGAGGAGATTCAGCCACAGTATGCTATC 1320

ALS-C AGGGAATTCCCCCTGGGGTACAAAACTTTTGATGAGGAGATTCAGCCACAGTATGCTATC 1320

***************************** ******************************

ALS-A CAGGTTCTGGATGAGCTGACGAAAGGGGAGGCCATCATTGCCACTGGTGTTGGGCAGCAC 1380

ALS-B CAGGTTCTGGATGAGCTGACCAAAGGGGAGGCCATCATTGCCACTGGTGTTGGGCAGCAC 1380

ALS-C CAGGTTCTGGATGAGCTGACGAAAGGGGAGGCCATCATTGCCACTGGTGTTGGGCAACAC 1380

******************** *********************************** ***

ALS-A CAGATGTGGGCGGCACAGTACTACACTTACAAGCGGCCAAGGCAGTGGTTGTCTTCAGCT 1440

ALS-B CAGATGTGGGCGGCACAGTACTACACTTACAAGCGGCCAAGGCAGTGGTTGTCTTCAGCT 1440

ALS-C CAGATGTGGGCGGCACAGTACTACACTTACAAGCGACCAAGGCAGTGGTTGTCTTCAGCT 1440

*********************************** ************************

ALS-A GGTCTTGGGGCTATGGGATTTGGTTTACCAGCTGCTGCTGGTGCTGCTGTGGCCAACCCA 1500

ALS-B GGTCTTGGGGCTATGGGATTTGGTTTGCCAGCTGCTGCTGGTGCTGCTGTGGCCAACCCA 1500

ALS-C GGTCTTGGAGCTATGGGATTTGGTTTGCCGGCTGCTGCTGGTGCTGCTGTGGCGAACCCA 1500

******** ***************** ** *********************** ******

ALS-A GGTGTTACAGTTGTTGACATCGATGGGGATGGCAGCTTCCTCGTGAACATTCAGGAGTTG 1560

ALS-B GGTGTTACAGTTGTTGACATCGATGGGGATGGCAGCTTCCTCATGAACATTCAGGAGTTG 1560

ALS-C GGTGTTACAGTTGTTGACATCGATGGGGATGGCAGCTTCCTCATGAACATTCAGGAGTTG 1560

**SubB_F** **AGTTG**

****************************************** *****************

ALS-A GCTATGATCCGCATTGAGAACCTCCCAGTGAAGGTCTTTGTGCTAAACAACCAACACCTG 1620

ALS-B GCTATGATCCGCAT**C**GAGAACCTCCCAGTGAAGGTCTTTGTGCTAAACAACCAACACCTG 1620

ALS-C GCTATGATCCGCATTGAGAACCTCCCAGTGAAGGTCTTTGTGCTAAACAACCAACACCTT 1620

**SubB_F** **GCTATGATCCGCATC**

************** ********************************************

ALS-A GGGATGGTGGTGCAGTGGGAGGACAGATTCTACAAGGCCAACCGAGCACACACATACTTG 1680

ALS-B GGTATGGTGGTGCAGTGGGAGGACAGATTCTACAAGGCCAACAGAGCACACACATACTTG 1680

ALS-C GGGATGGTGGTGCAGTGGGAGGACAGATTCTACAAGGCCAACCGAGCACATACATACTTG 1680

**SubA_F** **GAGCACACACATACTTG**

** *************************************** ******* *********

ALS-A GGGAA**T**CCAGAGAATGAGAGCGAGATATATCCGGATTTCGTGACGATTGCCAAAGGATTC 1740

ALS-B GGGAACCCAGAGAATGAGAGCGAGATATATCCGGATTTTGTAACGATTGCCAAAGGGTTC 1740

ALS-C GGGAACCCAGATAATGAGAGCGAGATATATCCGGATTTCGTGAC**C**ATTGCCAAAGGTTTT 1740

**SubA_F GGGCAT**

**SubC_F**  **GATATATCCGGATTTCGTGACC**

***** ***** ************************** ** ** *********** **

ALS-A AACATTCCAGCAGTCCGTGTGACAAAGAAGAGCGAAGTCCGTGCAGCAATTAAGAAGATG 1800

ALS-B AACATTCCAGCAGTCCGTGTGACAAAGAAGAGCGAAGTACGTGCAGCAATCAAGAAGATG 1800

ALS-C AACATTCCAGCGGTCCGTGTGACAAAGAAGAGCGAAGTACGTGCAGCAATCAAGAAGATG 1800

*********** ************************** *********** *********

ALS-A CTCGAGACTCCAGGGCCGTACCTGTTGGATATCATTGTCCCGCACCAGGAACATGTGTTG 1860

ALS-B CTCGAGACTCCAGGGCCGTACCTGTTGGATATCATTGTCCCGCACCAGGAACATGTGTTG 1860

ALS-C CTCGAGACTCCAGGGCCATACCTGTTGGATATCATTGTCCCGCACCAGGAACATGTGTTG 1860

***************** ******************************************

ALS-A CCTATGATCCCGAGCGGTGGCGCTTTCAAGGACATGATCCTGGATGGTGATGGCAGGACC 1920

ALS-B CCTATGATCCCGAGCGGTGGCGCTTTCAAGGACATGATCCTGGATGGTGATGGCAGGACC 1920

ALS-C CCTATGATCCCGAGCGGTGGCGCTTTCAAGGACATGATCCTGGATGGTGATGGCAGGACC 1920

************************************************************

ALS-A GTGTATTGA 1929

ALS-B GTGTATTGA 1929

ALS-C GTGTATTGA 1929

*********

Suppl. Fig. S1. Alignment of the whole *ALS* gene from three subgenomes showing the SNPs that differentiate them and specific primers targeting each one. The sequences are from Genbank: MH013494.1 (*ALS*-SubA), MG188321.1 (*ALS*-SubB), and MH013493.1 (*ALS*-SubC). Asterisk (*) indicates the same nucleotide in all three subgenomes. Absence of * indicates there is at least one different nucleotide between them.


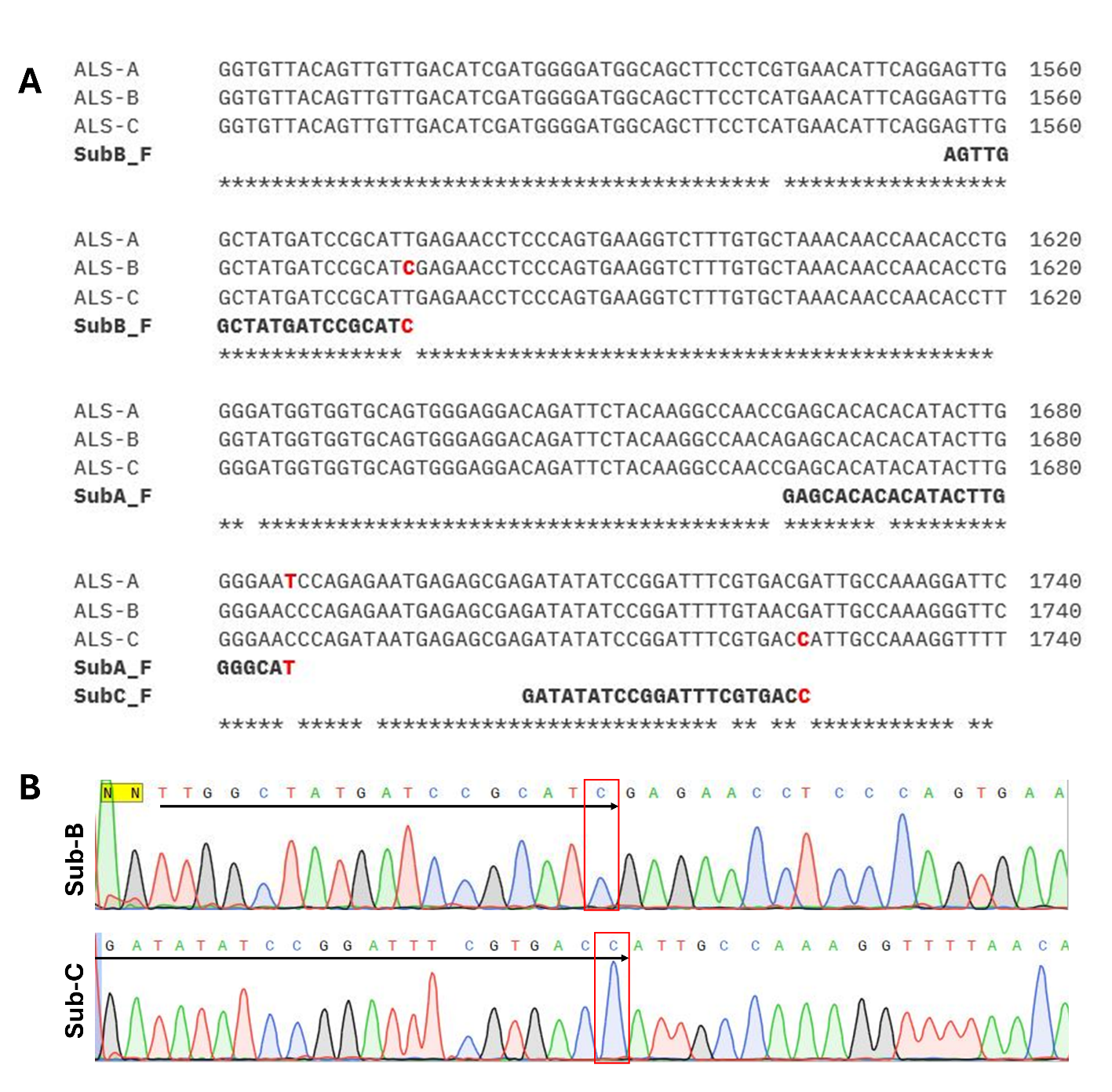


Suppl. Fig. S2. Confirmation of specificity of the primers targeting the three *ALS* from different subgenomes in expression analysis (A) Partial *E. crus-galli* *ALS* sequence showing the SNPs that differentiate them in each subgenome, and the forward primers targeting the SNPs at the 3’-end. (B) Sequencing of the PCR product when utilizing the *ALS*-specific primers targeting subgenome B or C, showing the absence of a double-peak at the primer 3’-end where the specific SNPs are.

Suppl. Table S1. Doses of four ALS inhibitors utilized for the dose-response curve with *E. crus-galli* biotypes with different *ALS* mutations

| Biotypes | Doses | Label rate range |
| --- | --- | --- |
| Imazethapyr | | |
| MOSTS-01 and CAPL-01 | 0; 3.312; 6.625; 13.25; 26.5; 53; 106; 212 g ha^-1^ | 0 - 2x |
| ARRGR-01 and PALMS-01 | 0; 13.25; 26.5; 53; 106; 212; 424; 848 g ha^-1^ | 0 - 8x |
| BAGÉ-01, SANTPAT-01, 423, CAMAQ-01, SAOJER-01, and CAPV-03 | 0; 53; 106; 212; 424; 848; 1,696; 3.392 g ha^-1^ | 0 - 32x |
| Penoxsulam | | |
| MOSTS-01 and CAPL-01 | 0; 0.6; 3.75; 7.5; 15; 30; 60; 120 g ha^-1^ | 0 - 2x |
| ARRGR-01, PALMS-01, BAGÉ-01, and SANTPAT-01 | 0; 3.75; 7.5; 15; 30; 45; 60; 120 g ha^-1^ | 0 - 2x |
| 423 | 0; 3.75; 7.5; 15; 30; 60; 120; 240 g ha^-1^ | 0 - 4x |
| CAPV-03 | 0; 15; 30; 60; 120; 240; 480; 960 g ha^-1^ | 0 - 16x |
| CAMAQ-01 and SAOJER-01 | 0; 20; 60; 180; 540; 1,620; 4,860; 14,580 g ha^-1^ | 0 - 243x |
| Bispyribac-sodium | | |
| MOSTS-01 and CAPL-01 | 0; 0.5; 3.125; 6.25; 12.5; 25; 50; 100 g ha^-1^ | 0 - 2x |
| ARRGR-01, PALMS-01, BAGÉ-01, and SANTPAT-01 | 0; 6.25; 12.5; 25; 37.5; 50; 100; 200 g ha^-1^ | 0 - 4x |
| 423 | 0; 3.125; 6.25; 12.5; 25; 50; 100; 200 g ha^-1^ | 0 - 4x |
| CAMAQ-01, SAOJER-01, and CAPV-03 | 0; 25; 50; 100; 200; 400; 800; 1,600 g ha^-1^ | 0 - 32x |
| Nicosulfuron | | |
| MOSTS-01 and CAPL-01 | 0; 0.6; 3.75; 7.5; 15; 30; 60; 120 g ha^-1^ | 0 - 2x |
| ARRGR-01, PALMS-01, BAGÉ-01, and SANTPAT-01 | 0; 7.5; 15; 30; 45; 60; 120; 240 g ha^-1^ | 0 - 4x |
| 423 | 0; 3.75; 7.5; 15; 30; 60; 120; 240 g ha^-1^ | 0 - 4x |
| CAMAQ-01, SAOJER-01, and CAPV-03 | 0; 30; 60; 120; 240; 480; 960; 1,920 g ha^-1^ | 0 - 32x |

Suppl. Table S2. Primers utilized as reference in the copy number variation and relative gene expression assays.

| Gene | Primer | Sequence (5' - 3') | Fragment size | Annealing T°C | Reference |
| --- | --- | --- | --- | --- | --- |
| *GAPDH* | GAPDH_F | TGGAATTGCTTTGAACGACA | 99 bp | 60 | (Iwakami et al., 2014) |
|  | GAPDH_R | AAGATGTGGCGGATCAGGT |  |  |  |
| *eIF4B* | eIF4B_F | GGGAAGTGATTTTGCAGGAG | 190 bp | 60 | (Iwakami et al., 2014) |
|  | eIF4B_R | GAGGCTTGGTCAGAACCATC |  |  |  |
| *RUB* | RUB_F | GGAGTATGAAACCAAGGATACTG | 100 bp | 60 | (Duhoux & Délye, 2013) |
|  | RUB_R | GTTGTCCATGTACCAGTAGAAGA |  |  |  |
| *18S* | 18S_F | GTGACGGAGAATTAGGGTTC | 100 bp | 60 | (Duhoux & Délye, 2013) |
|  | 18S_R | TGTCAGGATTGGGTAATTTG |  |  |  |
| *28S* | 28S_F | CTGATCTTCTGTGAAGGGT | 200 bp | 60 |  |
|  | 28S_R | TGATAGAACTCGTAATGGGC |  |  |  |

Suppl. Table S3. Screening of 100 *Echinochloa* populations from Southern Brazil with four ALS-inhibitor chemical groups: imazethapyr, penoxsualm, bispyribac-sodium, and nicosulfuron from imidazolinones, triazolopyrimidine – type 2, pyrimidinylbenzoates, and sulfonylureas, respectively. Efficacy of control 28 days after treatment (0% = no injuries; 100% = dead)

| ID | Municipality | Imazethapyr | Penoxsulam | Bispyribac-sodium | Nicosulfuron |
| --- | --- | --- | --- | --- | --- |
| 1 | CAMAQUÃ | 25.00 | 50.00 | 30.00 | 10.00 |
| 2 | TAPES | 21.67 | 100.00 | 100.00 | 95.00 |
| 3 | TAPES | 30.00 | 100.00 | 100.00 | 85.00 |
| 4 | TAPES | 75.00 | 81.67 | 58.33 | 75.00 |
| 5 | TAPES | 100.00 | 100.00 | 100.00 | 93.33 |
| 6 | TAPES | 48.33 | 23.33 | 40.00 | 50.00 |
| 7 | VENANCIO AIRES | 26.67 | 3.33 | 41.67 | 11.67 |
| 8 | SANTA CRUZ | 23.33 | 43.33 | 56.67 | 41.67 |
| 9 | SANTA CRUZ | 60.00 | 30.00 | 20.00 | 48.33 |
| 10 | SÃO PEDRO | 20.00 | 0.00 | 55.00 | 18.33 |
| 11 | SÃO PEDRO | 46.67 | 100.00 | 100.00 | 93.33 |
| 12 | SÃO PEDRO | 50.00 | 51.67 | 26.67 | 30.00 |
| 13 | AGUDO | 40.00 | 100.00 | 100.00 | 100.00 |
| 14 | PARAISO DO SUL | 41.67 | 0.00 | 31.67 | 10.00 |
| 15 | MOSTARDAS | 33.33 | 0.00 | 21.67 | 10.00 |
| 16 | CAPÃO DO LEAO | 50.00 | 100.00 | 100.00 | 100.00 |
| 17 | CAPÃO DO LEAO | 93.33 | 100.00 | 100.00 | 100.00 |
| 18 | CAPÃO DO LEAO | 18.33 | 100.00 | 95.00 | 93.33 |
| 19 | PIRATINI | 65.00 | 65.00 | 36.67 | 25.00 |
| 20 | PIRATINI | 43.33 | 0.00 | 25.00 | 20.00 |
| 21 | HULHA NEGRA | 53.33 | 21.67 | 53.33 | 50.00 |
| 22 | ACEGUÁ | 50.00 | 50.00 | 31.67 | 20.00 |
| 23 | BAGE | 50.00 | 100.00 | 91.67 | 100.00 |
| 24 | DOM PEDRITO | 48.33 | 15.00 | 25.00 | 15.00 |
| 25 | DOM PEDRITO | 31.67 | 21.67 | 36.67 | 10.00 |
| 26 | DOM PEDRITO | 21.67 | 100.00 | 100.00 | 90.00 |
| 27 | DOM PEDRITO | 56.67 | 0.00 | 15.00 | 0.00 |
| 28 | CHARQUEADAS | 60.00 | 100.00 | 100.00 | 100.00 |
| 29 | PANTANO GRANDE | 75.00 | 100.00 | 100.00 | 95.00 |
| 30 | RIO PARDO | 56.67 | 100.00 | 85.00 | 90.00 |
| 31 | MASSAMBARÁ | 100.00 | 100.00 | 100.00 | 100.00 |
| 32 | CAMAQUÃ | 50.00 | 46.67 | 30.00 | 35.00 |
| 33 | URUGUAIANA | 75.00 | 100.00 | 100.00 | 100.00 |
| 34 | URUGUAIANA | 100.00 | 100.00 | 100.00 | 100.00 |
| 35 | URUGUAIANA | 53.33 | 33.33 | 31.67 | 20.00 |
| 36 | ARROIO GRANDE | 100.00 | 100.00 | 100.00 | 100.00 |
| 37 | CAMAQUÃ | 48.33 | 100.00 | 100.00 | 100.00 |
| 38 | SANTA VITORIA DO PALMAR | 20.00 | 23.33 | 45.00 | 10.00 |
| 39 | URUGUAIANA | 46.67 | 35.00 | 33.33 | 16.67 |
| 40 | SANTA VITORIA DO PALMAR | 100.00 | 100.00 | 100.00 | 100.00 |
| 41 | TUBARÃO | 26.67 | 20.00 | 3.33 | 15.00 |
| 42 | IÇARA | 20.00 | 20.00 | 43.33 | 10.00 |
| 43 | MOSTARDAS | 35.00 | 26.67 | 10.00 | 1.67 |
| 44 | PELOTAS | 50.00 | 40.00 | 26.67 | 25.00 |
| 45 | ITAJAÍ | 55.00 | 53.33 | 33.33 | 70.00 |
| 46 | ARARANGUÁ | 18.33 | 20.00 | 45.00 | 0.00 |
| 47 | PELOTAS | 100.00 | 100.00 | 100.00 | 100.00 |
| 48 | CAMAQUÃ | 25.00 | 100.00 | 100.00 | 100.00 |
| 49 | CAMAQUÃ | 45.00 | 23.33 | 33.33 | 28.33 |
| 50 | CACHOEIRA DO SUL | 30.00 | 26.67 | 38.33 | 33.33 |
| 51 | RIO PARDO | 100.00 | 100.00 | 100.00 | 100.00 |
| 52 | TAPES | 32.50 | 93.33 | 100.00 | 100.00 |
| 53 | TAPES | 45.00 | 100.00 | 100.00 | 100.00 |
| 54 | VIAMAO | 100.00 | 100.00 | 100.00 | 100.00 |
| 55 | AGUDO | 43.33 | 20.00 | 45.00 | 43.33 |
| 56 | ITAQUI | 10.00 | 20.00 | 35.00 | 13.33 |
| 57 | RIO GRANDE | 23.33 | 22.50 | 66.67 | 31.67 |
| 58 | MOSTARDAS | 100.00 | 100.00 | 100.00 | 100.00 |
| 59 | CACHOEIRA DO SUL | 100.00 | 100.00 | 100.00 | 100.00 |
| 60 | MOSTARDAS | 100.00 | 100.00 | 100.00 | 100.00 |
| 61 | CACHOEIRINHA | 100.00 | 100.00 | 100.00 | 100.00 |
| 62 | CACHOEIRINHA | 100.00 | 100.00 | 100.00 | 100.00 |
| 63 | CACHOEIRINHA | 100.00 | 100.00 | 100.00 | 100.00 |
| 64 | SANTA MARIA-01 | 100.00 | 100.00 | 100.00 | 100.00 |
| 65 | ANTA GORDA-01 | 100.00 | 100.00 | 100.00 | 100.00 |
| 66 | CAPAO DO LEÃO | 100.00 | 100.00 | 100.00 | 100.00 |
| 67 | PORTO ALEGRE | 100.00 | 100.00 | 100.00 | 100.00 |
| 68 | VALE VERDE | 100.00 | 100.00 | 100.00 | 100.00 |
| 69 | BAGE | 30.00 | 100.00 | 100.00 | 100.00 |
| 70 | PALMARES DO SUL | 30.00 | 100.00 | 100.00 | 100.00 |
| 71 | MOSTARDAS | 43.33 | 50.00 | 50.00 | 20.00 |
| 72 | CAMAQUÃ | 50.00 | 70.00 | 51.67 | 36.67 |
| 73 | RIO GRANDE | 40.00 | 50.00 | 35.00 | 33.33 |
| 74 | RIO PARDO | 40.00 | 40.00 | 40.00 | 23.33 |
| 75 | PALMARES DO SUL | 63.33 | 100.00 | 100.00 | 100.00 |
| 76 | CAPOVARI DO SUL | 40.00 | 38.33 | 30.00 | 23.33 |
| 77 | SÃO JERONIMO | 10.00 | 30.00 | 0.00 | 23.33 |
| 78 | SANTO ANTONIO DA PATRULHA | 20.00 | 100.00 | 100.00 | 100.00 |
| 79 | ARROIO GRANDE | 33.33 | 100.00 | 100.00 | 100.00 |
| 80 | CACHOEIRA DO SUL | 75.00 | 100.00 | 100.00 | 100.00 |
| 81 | ARROIO GRANDE | 65.00 | 100.00 | 100.00 | 100.00 |
| 82 | ARROIO GRANDE | 73.33 | 100.00 | 100.00 | 100.00 |
| 83 | CACHOEIRA DO SUL | 33.33 | 100.00 | 100.00 | 100.00 |
| 84 | CANDELÁRIA | 30.00 | 100.00 | 100.00 | 100.00 |
| 85 | CANDELÁRIA | 33.33 | 100.00 | 100.00 | 100.00 |
| 86 | CACHOEIRA DO SUL | 50.00 | 52.50 | 50.00 | 56.67 |
| 87 | AGUDO | 20.00 | 100.00 | 100.00 | 100.00 |
| 88 | ARROIO GRANDE | 100.00 | 100.00 | 100.00 | 100.00 |
| 89 | CERRITO | 18.33 | 100.00 | 100.00 | 100.00 |
| 90 | CERRITO | 30.00 | 100.00 | 100.00 | 100.00 |
| 91 | MOSTARDAS | 33.33 | 96.67 | 100.00 | 100.00 |
| 92 | PIRATINI | 31.67 | 3.33 | 0.00 | 15.00 |
| 93 | PIRATINI | 25.00 | 26.67 | 18.33 | 21.00 |
| 94 | HULHA NEGRA | 20.00 | 36.67 | 25.00 | 25.00 |
| 95 | DOM PEDRITO | 6.67 | 33.33 | 45.00 | 35.00 |
| 96 | ITAQUI | 0.00 | 6.67 | 10.00 | 12.00 |
| 97 | ITAQUI | 3.33 | 5.00 | 26.67 | 18.00 |
| 98 | ITAQUI | 13.33 | 0.00 | 25.00 | 30.00 |
| 99 | URUGUAIANA | 0.00 | 0.00 | 23.33 | 14.00 |
| 100 | CACEQUI | 51.67 | 66.67 | 36.67 | 35.00 |

Suppl. Table S4. Dose-response curve to penoxsulam utilizing biomass (g) and herbicide efficacy (%) variables of two *E. crus-galli* biotypes with Trp574 mutation in *ALS* gene (CAMAQ-01 and SAOJER-01), and one susceptible without mutation (MOSTS-01)

| Biotypes | Mutation | *b* | *d* | *e*(GR_50_ or ED_50_) | GR_50_ or ED_50_ Lower limit | GR_50_ or ED_50_ Upper limit | RF |
| --- | --- | --- | --- | --- | --- | --- | --- |
|  |  | Biomass (g) | | | | | |
| MOSTS-01 | No | 0.93 ^ns^ | 5.98* | 0.04 ^ns^ | -0.14 | 0.23 | - |
| CAMAQ-01 | Trp574Leu | 0.78* | 3.27* | 54.78* | 12.50 | 97.05 | 1369.4 |
| SAOJER-01 | Trp574Leu | 0.79* | 4.14* | 1231.32* | 597.30 | 1865.34 | 30783.0 |
|  |  | Herbicide efficacy (%) | | | | | |
| MOSTS-01 | No | -2.19^ns^ | 100.06* | 0.27^ns^ | -0.49 | 1.04 | - |
| CAMAQ-01 | Trp574Leu | -1.09* | 101.06* | 263.67* | 209.91 | 317.44 | 976.6 |
| SAOJER-01 | Trp574Leu | -1.25* | 58.09* | 4457.42* | 580.7 | 8334.14 | 16509.0 |

*significant parameter (p<0.05); ^ns^non-significant parameter; RF = Resistance factor; GR_50_: herbicide dose that reduced 50% of the dry biomass; ED_50_: herbicide dose that achieved 50% in control efficacy.
